# Greater pain and functional impairment in chronic erosive hand osteoarthritis compared to treated rheumatoid arthritis: A comparative study

**DOI:** 10.1016/j.ocarto.2025.100626

**Published:** 2025-05-20

**Authors:** S. Berkani, S. Tuffet, A. Rousseau, N. Rincheval, E. Maheu, B. Combes, A. Saraux, B. Fautrel, L. Gossec, F. Berenbaum, J. Sellam, A. Courties

**Affiliations:** a Sorbonne Université, Assistance Publique – Hôpitaux de Paris (AP-HP), Centre de Recherche Saint-Antoine Inserm UMRS_938, Saint-Antoine Hospital, Department of Rheumatology, Paris, France; b Department of Clinical Pharmacology and Clinical Research Platform Paris-East (URCEST-CRC-CRB), AP-HP, Saint-Antoine Hospital, Paris, France; c Department of Rheumatology, University of Montpellier, Montpellier, France; d Rheumatology Department, CHRU de Brest, Site Cavale Blanche, Brest, France; e Inserm, LabEx IGO, UMR1227, Lymphocytes B et Autoimmunité, Université de Bretagne Occidentale, Brest, France; f Sorbonne-Université, INSERM, Institut Pierre Louis d’Epidémiologie et de Santé Publique, Paris France; g AP-HP, Pitié-Salpêtrière Hospital, Rheumatology department, Paris, France; h Inserm UMRS_938, Centre de Recherche Saint-Antoine, Paris, France

**Keywords:** Hand osteoarthritis, Rheumatoid arthritis, Burden disease, Comorbidities

## Abstract

**Objectives:**

To compare the burden of established chronic rheumatoid arthritis (RA) and erosive hand osteoarthritis (EHOA), in terms of pain, functional impairment, comorbidities, and cardiometabolic diseases (CMD).

**Methods:**

This study included EHOA patients from the inclusion visit of the DIGICOD cohort and RA patients from the 10th year visit of the ESPOIR cohort. Outcomes were pain intensity (≥40 on a 0–100 ​mm visual analog scale [VAS]), VAS fatigue, and functional impairment, measured by normalized (0–100) Health Assessment Questionnaire (HAQ) for RA and the AUStralian CANadian Osteoarthritis Hand (AUSCAN) function for EHOA and binarized by their medians. Comorbidities and CMD were also assessed. Logistic regression models adjusted for age, sex, body mass index, and socio-educational level were used to compare outcomes.

**Results:**

We included 138 EHOA and 379 RA patients. EHOA patients were older (median age 67.3 *vs.* 48.6 years, p ​< ​0.001). EHOA patients were more likely to experience higher pain at mobilization (OR ​= ​3.13 95 ​% CI [1.74 to 5.68]) and greater functional impairment (OR ​= ​2.27, 95 ​% CI [1.26 to 4.17]) than RA patients. There was no difference for fatigue and pain at rest. The overall risk of comorbidities was lower in EHOA patients in multivariate analysis (OR ​= ​0.25, 95%CI [0.13–0.48]). There was no significant difference in CMD risk.

**Conclusion:**

After more than 10 years of disease duration, EHOA is associated with greater pain and functional impairment than treated RA but with fewer comorbidities. This highlights the significant unmet need for effective therapies for EHOA patients.

## 1 Introduction

Osteoarthritis (OA) is the most common musculoskeletal disease. It is often perceived as the least severe among these conditions and is associated with the inevitable articular aging [1]. Hand OA (HOA) is one of the most prevalent site of OA, affecting approximately one in two women and one in four men during their lifetime [2]. Patients with HOA experience a significant impact on their quality of life, with difficulties in carrying heavy objects, dressing, and eating [3,4]. Erosive HOA (EHOA), defined by the presence of central radiographic erosions [5], is characterized by higher levels of inflammatory markers, increased synovitis, and greater radiographic progression compared to non-erosive HOA [[6], [7], [8], [9], [10]]. Its affects about 10 ​% of patients with symptomatic HOA but can be present in 40–50 ​% of patients seen in tertiary care centers [11,12]. Individuals with EHOA are more likely to develop symptoms than those with non-erosive HOA (54.2 ​% vs. 28.3 ​%) and tend to experience more pain and disability [13,14]. As for all OA patients, the burden of the disease is also due to the high prevalence of comorbidities, particularly cardiometabolic conditions such as hypertension, metabolic syndrome or cardiovascular diseases [15]. The impact of comorbidities might be especially relevant in EHOA since some studies suggest a higher prevalence of cardiometabolic comorbidities in EHOA than in non-EHOA [16]. Comorbidities limit treatment options that are already quite scarce [17]. EHOA is therefore considered as a challenging disease to treat with a clear unmet need, whereas it is considered by patients and doctors as a “wear and tear” disease and part of “normal aging” [[18], [19], [20]].

In contrast, RA is the benchmark of severe rheumatic disease considered with a worse functional prognosis, more comorbidities, and a greater global burden than OA. In the 1980's, 92 ​% of patients had an important decrease of functional capacity and 50 ​% of them needed personal help of activities of daily living, at 10 years of evolution of the disease without treatment [21,22]. Because of the pain, the functional and psychological impact, RA patients have a reduced quality of life compared to the general population [[23], [24], [25], [26]]. Furthermore, the cardiovascular risk in rheumatoid arthritis (RA) is well-known and is related not only to increased cardiovascular risks but also to systemic inflammation and the adverse effects of treatments [27].

This paradigm distinguishing severe (i.e, RA) and non-severe (i.e, EHOA) rheumatic diseases may no longer be true with the advent of biologics and targeted therapies in RA, as the “treat-to-target” strategy has led to a reduction in disease activity over time, incidence of erosions, and comorbidities, as well as an improvement in patients' quality of life [28]. A recent study showed that at diagnosis, HOA appears as disabling as untreated RA [29]. Due to the lack of as effective treatments as in RA and its inflammatory phenotype, the burden of EHOA may now be as high or even higher that of treated RA. However, few studies have compared the burden of EHOA and RA, with heterogeneous outcomes (pain, stiffness, function, or quality of life) and populations (active and non-active RA; EHOA and HOA), limiting the generalizability of conclusions [17,[30], [31], [32]]. For example, one study suggests that both HOA and treated RA have similar levels of pain while another study contradicts this finding, suggesting that RA may be associated with lower levels of pain compared to both erosive and non-erosive HOA [6,32].

Since EHOA appeared the most severe and inflammatory form of OA, we aimed to compare the burdens of chronic EHOA and RA (as a benchmark for high burden disease) using two large French national cohorts: the early RA cohort (*Étude et Suivi des Polyarthrites Indifférenciées Récentes* (ESPOIR)) [33,34] and the HOA cohort (DIGICOD) [12]. Our objectives were to compare pain and functional impairment and the risk of associated comorbidities and cardiometabolic diseases (CMD).

## 2 Material and methods

### 2.1 Study participants

#### 2.1.1 Patients with EHOA from DIGICOD cohort

The DIGICOD cohort (NCT 01831570) includes patients aged 35 years and older, with symptomatic HOA (pain or nodosity) in two joints among distal or proximal interphalangeals (IP) joint or with thumb base OA and with radiographic stage according to Kellgren-Lawrence (KL) ​≥ ​2 [12,35,36]. Here, we included EHOA patients, defined by at least one erosive joint (E or R) according to the Verbruggen score [5] at the inclusion visit of DIGICOD cohort, because they already had a mean duration of the disease of more than 10 years. Ethical approval was obtained from the ethics committee Paris Île-de-France IV ethics committee (EUDRA-CT 2012-A01004-39, ref 2012/43C).

#### 2.1.2 Patients with treated RA form ESPOIR cohort

The ESPOIR baseline cohort includes patients between 18 and 70 years of age with confirmed or suspected RA or undifferentiated arthritis which may evolve to RA (NCT 03666091). Inclusion criteria were at least two swollen joints since at least 6 weeks and less than 6 months without prior treatment. Patients with ACR/EULAR 2010 RA criteria [37] who completed the 10th year visit of ESPOIR cohort were included. The protocol of the ESPOIR Cohort study was approved in July 2002 by the ethical committee of Montpellier (N° 020307).

#### 2.1.3 Data collection

Data extracted from the two cohorts were patient's demographic characteristics at the time of inclusion in the study including sex, age, body mass index, socio-educational level (higher, secondary and primary education), smoking and alcohol status, medical history of high blood pressure, dyslipidemia, myocardial ischemia, stroke, diabetes, neoplasia, blood disease, chronic hepatitis B or C, vertebral fracture and other fractures. We also collected disease characteristics with disease duration, therapeutics (Dmards, corticosteroids, NSAIDs, opioids), number of painful and swollen joints on the hands (including wrist, metacarpophalangeal, proximal interphalangeal, for RA and EHOA, adding distal interphalangeal for EHOA), levels of C-reactive protein (CRP), and disease activity score on 28 joints (DAS-28 CRP) rheumatoid factor, ACPA, Sharp-van der Heijde score [38]) for RA patients. Patients reported outcomes with visual analog scale (VAS) pain at rest, VAS pain at mobilization, VAS fatigue, function (Health Assessment questionnaire (HAQ) for RA patients and Australian/Canadian OA hand index (AUSCAN) function for EHOA patients) were also extracted. Patients with missing data for outcomes or for the adjustment variables were excluded.

#### 2.1.4 Outcomes

Clinical burden was defined as a VAS pain at mobilization ≥40/100 (considered as patient acceptable symptom state [39]), VAS pain at rest ≥40/100, VAS fatigue ​≥ ​median and normalized functional score ​≥ ​median. Functional scores were HAQ normalized to 100 for RA and AUStralian CANadian Osteoarthritis Hand function normalized to 100 for EHOA.

We defined cardiometabolic comorbidities as a medical history of HBP, diabetes, dyslipidemia, myocardial infarction or stroke. To compare the burden of comorbidities, we assessed the risk of having at least two comorbidities (among CMD, cancer, hemopathy, fracture) and the risk of having at least one CMD.

#### 2.1.5 Statistical analysis

Continuous variables are described by median and interquartile range, based on their distributions (checked graphically on histograms). Categorical variables are described by their number and frequency. Categorical data were compared using Fisher's exact test and continuous variable were compared using the Wilcoxon non-parametric rank test. Since the age distribution differs significantly between the two populations, we evaluated each outcome by age group. Associations between outcomes and EHOA and RA populations were studied using logistic regression models. Unadjusted and adjusted analyses were performed. Adjustment was made for main common determinants of pain in both diseases: age, sex, BMI and socio-educational level. For clinical burden, additional adjustment was made for the number of comorbidities. Results are presented as odds ratios (OR) and their 95 ​% confidence intervals (CI), considering EHOA versus RA as reference. For each model, goodness of fit was checked using Hosmer-Lemeshow test and diagnostics of residuals were studied. Sensitivity analyses after exclusion of influential individuals were performed to check the robustness of the models.

## 3 Results

### 3.1 Demographic and clinical characteristics

**Fig. 1:**
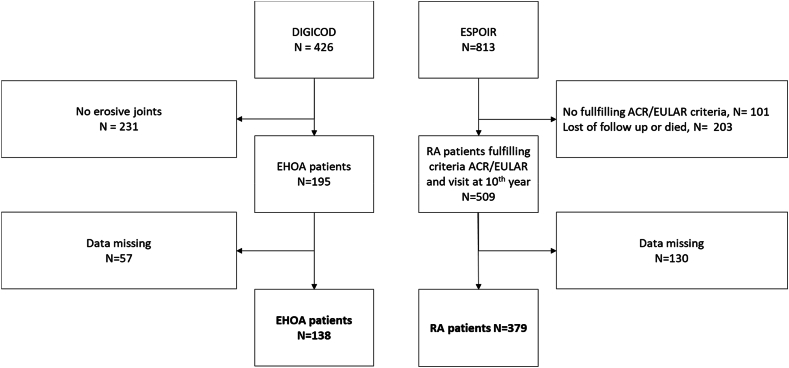
Inclusion flow chart of patients. EHOA ​= ​erosive hand osteoarthritis; RA ​= ​Rheumatoid arthritis.

From the 426 patients included at the baseline visit of the DIGICOD cohort, 195 had EHOA and among them 138 patients were analyzed because of missing data. From the 813 patients included at baseline in the ESPOIR cohort, 379 patients who met the 2010 ACR/EULAR criteria for RA and attended the 10th-year visit (Fig. 1) were analyzable. The EHOA patients were older than RA patients with a median [IQR] age of 67 [64–72] *vs*. 49 years [39–56] (p ​< ​0.001). The median disease duration was 13.5 [7.0–20.0] years for EHOA patients *versus* 10.5 [10.3–10.7] years for RA patients (p ​= ​0.003). The median disease duration of all patients was 10.5 [10.3–11.1].

RA patients were more likely to smoke (16.6 ​% vs. 6.5 ​%, p ​< ​0.05) than EHOA patients, but less likely to drink alcohol (15.8 ​% *vs.* 76.8 ​%). For comorbidities: EHOA had more frequent high blood pressure than RA (64.5 ​% *versus* 29.3 ​%, p ​< ​0.001), while other CMD were not different (Table 1).

**Table 1:** Characteristics of patients.

	EHOA (n ​= ​138)	RA (n ​= ​379)	P value
Female, n (%)	113 (81.9 ​%)	289 (76.3 ​%)	0.174
Years, median (IQR)	67.3 (64.3, 72.2)	48.6 (39.3, 55.6)	<0.001
Body mass index, median (IQR)	24.8 (22.2, 27.3)	25.5 (22.4, 28.7)	<0.001
Socio educational level, n (%)			<0.001
Higher education	77 (55.8 ​%)	344 (90.8 ​%)	–
Secondary education	45 (32.6 ​%)	0 (0 ​%)	–
Primary education	16 (11.6 ​%)	35 (9.2 ​%)	–
Current smoker, n (%)	9 (6.5 ​%)	63 (16.6 ​%)	0.002
Current alcohol consumption, n (%)	106 (76.8 ​%)	60 (15.8 ​%)	<0.001
Disease duration, median year (IQR)	13.5 (7.0, 20.0)	10.5 (10.3, 10.7)	0.003
RF IgM-positive, n (%)	7 (5.1 ​%)	219 (57.8 ​%)	<0.001
ACPA positivity, n (%)	–	206 (54.4 ​%)	–
Sharp-van der Heijde score, median (IQR)^a^	–	5.3 (2.0, 14.8)	–
DAS 28 CRP, median (IQR)^b^	–	2.3 (1.6, 3.2)	–
CRP ≥ 5 ​mg/L, n (%)^c^	11 (8.0 ​%)	119 (32.2 ​%)	<0.001
Treatments, n (%)			
Salazopyrine, Leflunomide or Hydroxchloroquine	–	179 (47.2 ​%)	–
Methotrexate	–	310 (81.8 ​%)	–
Biological Dmards	–	140 (36.9 ​%)	–
Glucorcorticoids during the past year, n(%)^d^	2 (1.4 ​%)	96 (25.3 ​%)	<0.001
Mean dose a day, median (IQR)^d^	1.5 (1.3, 1.8)	4 (1.1, 5.0)	0.301
Non steroidal anti inflammatory drugs, n (%) (%)	28 (20.3 ​%)	128 (33.8 ​%)	0.003
Opioids, n (%)	–	1 (0.3 ​%)	–
Number of comorbidities, n (%)			0.002
0	33 (23.9 ​%)	143 (37.7 ​%)	
1	53 (38.4 ​%)	132 (34.8 ​%)	
2	40 (29.0 ​%)	62 (16.4 ​%)	
≥ 3	12 (8.7 ​%)	42 (11.1 ​%)	
Hepatitis virus B or C, n (%)	1 (0.7 ​%)	8 (2.1 ​%)	0.456
High blood pressure, n (%)	89 (64.5 ​%)	111 (29.3 ​%)	<0.001
Dyslipidemia, n (%)	52 (37.7 ​%)	155 (40.9 ​%)	0.543
Diabete, n (%)	10 (7.2 ​%)	34 (9.0 ​%)	0.600
Myocardial infarction, n (%)	1 (0.7 ​%)	11 (2.9 ​%)	0.196
Stroke, n (%)	4 (2.9 ​%)	7 (1.8 ​%)	0.495
Neoplasia, n (%)	16 (11.6 ​%)	39 (10.3 ​%)	0.747
Hemopathy, n (%)	0 (0.0 ​%)	4 (1.1 ​%)	0.578
Spine fracture, n (%)	0 (0.0 ​%)	7 (1.8 ​%)	0.180
Other fracture, n (%)	0 (0.0 ​%)	31 (8.2 ​%)	<0.001

EHOA ​= ​erosive hand osteoarthritis; RA ​= ​Rheumatoid arthritis. Data are expressed as number (percent) or median and interquartile interval. P values are Tisher's exact test or Wilcoxon rank test, depending on the variable. Missing value in the RA population.

a N ​= ​87.

b N ​= ​28.

c N ​= ​10.

d N ​= ​283.

### 3.2 Pain and functional outcomes

**Fig. 2:**
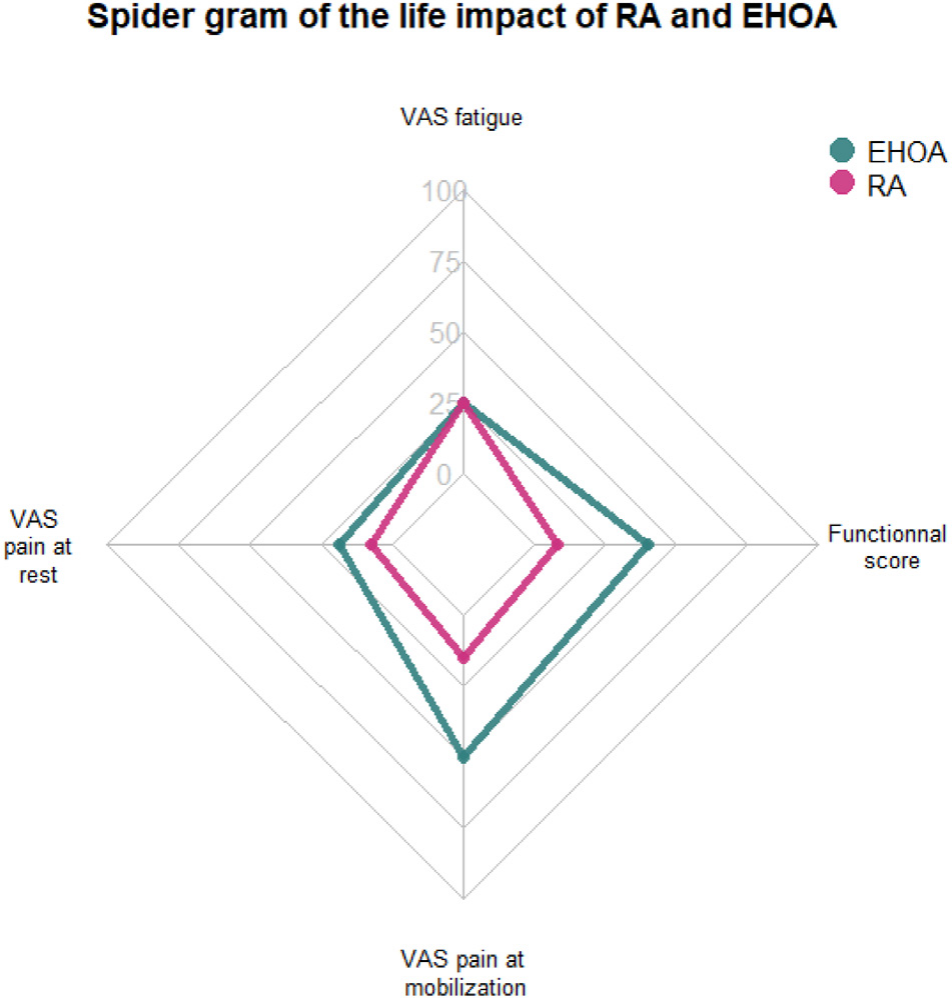
Spidergram of the life impact of RA and EHOA. VAS ​= ​visual analogscale, EHOA ​= ​erosive hand osteoarthritis, RA ​= ​rheumatoid arthritis.

EHOA patients had 2 painful joints [1.0–3.5] and 4 swollen joints [2.0–3.8] in the hands, whereas RA patients had 0 painful [0.0–2.0] and 0 swollen joints [0.0–3.0] (p ​< ​0.001). VAS pain at rest and mobilization were higher in EHOA than in RA, with a median [IQR] respectively of 18.5 [0.0–40.8] *vs*. 7.0 [0.0–25.5] and 50 [23.2–70.0] *vs* 15 [2.0–40.0], respectively (Fig. 2). The normalized functional score was 40.0 [19.0–60.8] for EHOA and 8.3 [0.0–25] on a scale of 0–100 for RA patients (Table 2). Among patients aged 51–70 years — the largest overlapping age group — pain, functional impairment, and fatigue (VAS) remained higher in EHOA patients (Table 3). Notably, younger RA patients appeared to have milder disease activity.

**Table 2:** Results of pain and functional outcomes.

	EHOA	RA	Unadjusted		Adjusted	
	N=138 Median (IQR)	N=379 Median (IQR)	OR (CI 95 ​%) EHOA (*versus* RA)	P value	OR (CI 95 ​%) EHOA (*versus* RA)	P value
VAS pain at rest (mm) ≥ 40/100	18.5 (0.0, 40.8)	7.0 (0.0, 25.5)	1.77 (1.10, 2.81)	0.016	1.64 (0.83, 3.24)	0.151
VAS pain at mobilization (mm) ≥ 40/100	50 (23.2, 70.0)	15 (2.0, 40.0)	4.03 (2.69, 6.09)	<0.001	3.13 (1.74, 5.68)	<0.001
VAS fatigue (mm) ≥ 25/100	25 (13.0, 47.0)	25 (5.5, 50.0)	1.15 (0.78, 1.70)	0.483	1.46 (0.84, 2.55)	0.163
Functional score ≥ 16.7/100	40.0 (19.0, 60.8)	8.3 (0.0, 25.0)	4.04 (2.63, 6.35)	<0.001	2.27 (1.26, 4.17)	0.007

VAS ​= ​visual analog scale; EHOA ​= ​erosive hand osteoarthritis; RA ​= ​rheumatoid arthritis.

**Table 3:** Results of pain and functional outcomes by class of age.

	EHOA		RA	
	N∗	Median (IQR)	N∗	Median (IQR)
Range by age				
VAS pain at rest (mm)				
< 50 years	1	–	205	5.0 (0.0; 23.0)
51–70 years	86	18.5 (0.0; 36.0)	172	9.0 (1.0; 29.5)
71–80 years	51	20.0 (0.0; 45.0)	2	–
VAS pain at mobilization (mm)				
< 50 years	1	–	205	12.0 (1.0; 40.0)
51–70 years	86	49.0 (22.0; 68.0)	172	21.5 (3.0; 42.0)
71–80 years	51	54.0 (27.0; 74.0)	2	–
VAS fatigue (mm)				
< 50 years	1	–	205	25.0 (5.0; 57.0)
51–70 years	86	30.0 (11.0; 50.0)	172	23.0 (6.0; 45.0)
71–80 years	51	22.0 (13.0; 44.0)	2	–
Functional score				
< 50 years	1	–	205	4.2 (0.0; 25.0)
51–70 years	86	38.0 (18.0; 61.0)	172	16.7 (0.0; 29.2)
71–80 years	51	41.0 (19.0; 59.0)	2	–

VAS ​= ​visual analog scale, EHOA ​= ​erosive hand osteoarthritis, RA ​= ​rheumatoid arthritis, N∗ ​= ​Total number of patient in the range of age.

In the unadjusted analysis, chronic EHOA was associated with a higher risk of VAS pain at rest ≥40/100 ​mm, and at mobilization ≥40/100 ​mm (OR: 1.77 [1.10, 2.81] p ​< ​0.05; OR: 4.03 [ 95 ​% CI 2.69–6.09], p ​< ​0.001) and a higher functional score than chronic RA. In adjusted analysis, the risk to have VAS pain at mobilization ≥40/100 ​mm and high functional scores remained significantly higher in EHOA patients than in RA patients, regardless of age, sex, BMI, comorbidities and socio-educational level. In fact, patients with EHOA were 3 times more likely to have VAS pain at mobilization greater than 40/100, as defined by the patient acceptable symptom state (OR: 3.13 [1.74–5.68], p ​< ​0.001). VAS pain at rest and fatigue were not modified after adjustment (Table 2 and Fig. 2).

### 3.3 Comorbidities outcomes

Overall, 38 ​% of EHOA patients had at least two comorbidities compared to 28 ​% of RA patients. However, given the significant age disparity between the groups, we analyzed the prevalence by age group. Between 50 and 70 years, 30 ​% of EHOA patients had at least two comorbidities compared to 47 ​% of RA patients (Table 4).

**Table 4:** Prevalence of patients with at least two comorbidities disease by class of age.

	EHOA		RA	
	N∗	n (%)	N∗	n (%)
Total	138	52 (38 ​%)	379	104 (27 ​%)
Range by age				
< 50 years	1	0 (0.0 ​%)	205	23 (11.2 ​%)
51–70 years	86	26 (30.2 ​%)	172	81 (47.1 ​%)
71–80 years	51	26 (51.0 ​%)	2	0 (0.0 ​%)

EHOA ​= ​erosive hand osteoarthritis, RA ​= ​rheumatoid arthritis, N∗ ​= ​Total number of patient in the range of age.

In the unadjusted analysis, the risk of having at least two comorbidities was higher in EHOA than in RA with an OR of 1.60 [ 95 ​% CI 1.06–2.41], p ​< ​0.001 as well as the risk of having one CMD with an OR of 2.48 [95 ​% CI 1.63–3.86], p ​< ​0.001. However, after adjustment for age, sex, BMI and socio–educational level, EHOA was associated with a lower risk of having two comorbidities, (OR 0.25 [95 ​% CI [0.13–0.48], p ​< ​0.001). There was no difference for the risk of having one CMD OR 0.57 [95 ​% CI 0.30–1.05] p ​= ​0.073] (Table 5).

**Table 5:** Comorbidites outcomes: prevalence, unadjusted and adjusted analyses.

	EHOA	RA	Unadjusted		Adjusted	
	N=138 n (%)	N=379 n (%)	OR (CI 95 ​%) EHOA (*versus* RA)	P value	OR (CI 95 ​%) EHOA (*versus* RA)	P value
> 2 comorbidities	52 (38 ​%)	104 (27 ​%)	1.60 (1.06, 2.41)	0.025	0.25 (0.13, 0.48)	<0.001
> 1 CMD	102 (73.9 ​%)	202 (53.3 ​%)	2.48 (1.63, 3.86)	<0.001	0.57 (0.30, 1.05)	0.073

Comparison using unadjusted and adjusted logistic regression models. Adjustment was made on age, sex, body mass index and socio educational level.

VAS ​= ​visual analog scale, EHOA ​= ​erosive hand osteoarthritis, RA ​= ​rheumatoid arthritis, CMD ​= ​cardiometabolic diseases.

## 4 Discussion

Our study highlights significant differences in the burden of chronic EHOA and RA. After more than 10 years of disease duration, patients with EHOA have a threefold higher risk of experiencing high level of pain and a twofold higher risk of high functional impairment compared to patients with chronic treated RA. Although the overall prevalence of comorbidities is higher in EHOA, the adjusted risk of having two comorbidities is four times lower in established EHOA than in treated chronic RA patients, while the risk of cardiometabolic disease is not different.

Our results are consistent with those of a cross-sectional study that included 167 EHOA and 79 patients with inflammatory arthritis (RA or psoriatic arthritis) patients with low disease activity (DAS28 ​< ​3.2). Patients with EHOA experienced more pain than patients with controlled inflammatory arthritis, with a mean difference in VAS pain of 14.8 ​mm on a scale of 0–100 ​mm (p ​< ​0.001) and more functional impairment according to the Functional Index for HOA and the AUStralian CANadian Osteoarthritis Hand [6]. Similar results were found in studies comparing RA and HOA (both erosive and non-erosive). In fact, two studies compared patients with RA and OA or HOA using the Multidimensional Health Assessment Questionnaire/Routine Assessment of Patient Index Data 3 (MDHAQ/RAPID3). RAPID3 is composed of 3 MDHAQ scores for physical function, pain VAS, and patient global assessment VAS [40]. In El-Haddad et al., 1157 patients were independently analyzed at four geographically different sites, including 531 with RA and 626 with OA. The median RAPID3 was significantly higher in OA than in RA at three sites, although the median disease duration was longer in the RA group than in the OA group. The results were adjusted for age, education level and disease duration [31]. A more recent study, including 149 HOA and 50 DMARD-naive RA patients, compared the two populations at the initial visit and at 6 months. The RAPID3 was higher in HOA patients than in DMARD-naïve RA patients. The RAPID3 decreased in both groups between the initial visit and 6 months, but the mean decrease in RAPID3 was higher in RA than in HOA [29]. In other words, RA patients improved more significantly than HOA patients, reflecting the effect of treatment and medical management.

Studies considering disease activity support this explanation. For example, in the study by Almeida et al. patients with HOA had the same level of pain and disability (HAQ) as patients with active RA and a higher level of pain than patients with RA in remission [30]. A study including 199 HOA and 194 RA patients suggested different results, showing a similar impact on health-related quality of life between RA and HOA patients assessed by the Short Form-36 (SF-36). Physical function was more altered in RA patients, but HOA patients reported worse higher pain scores on the Arthritis Impact Measurement Scale 2 [32]. The differences between this study and ours, regarding functional scores may be due to more severe structural damage in RA patients in the early 2000s, with limited availability of biological DMARDs, compared to those included at the 10-year visit of the ESPOIR cohort. Indeed, patients in the ESPOIR cohort, received exemplary care, as evidenced by a median DAS-28 score of 2.3, and a significantly lower number of swollen and tender joints.

All of these findings suggest that the introduction of bDMARDs, which allow clinical and structural remission of RA, has significantly reduced its burden, challenging the notion that RA remains a high-burden disease compared to HOA, particularly EHOA. We do not suggest that HOA should be compared to RA as “more severe” on a group or individual level; however, this underscores the urgent need for research and consideration in HOA to develop new effective therapeutic treatments (ie symptomatic as well as disease-modifying osteoarthritis drug). Along this line, some recent randomized controlled trial have given some valuable results [41].

The increased risk of comorbidities in EHOA found in the unadjusted analysis is probably mainly explained by the age difference between the two groups. Adjusted analyses show that EHOA patients have a significantly lower risk of cumulative comorbidities but a similar risk for cardiometabolic diseases (with a trend in favor of RA, p ​= ​0.07). The non-significant trend for cardiometabolic disease may be due to a lack of power because of the small number of cardiovascular events. The incidence of hypertension in patients with EHOA is twice as high as in patients with RA. In a recent meta-analysis, the prevalence of hypertension was found to be associated with OA, which warrants further detailed analysis [15]. Pro-inflammatory cytokines are increased in OA but less pronounced than in RA [42]. The association between OA and cardiovascular risk factors such as diabetes and dyslipidemia is well known but probably underestimated [43]. According to the European Alliance of Associations for rheumatology, cardiovascular risk prediction models for RA should be adjusted by a 1.5 multiplication factor, if this is not already included in the risk algorithm [44]. A recent study including 35 RA and 35 HOA (only 5 with the erosive form) showed no difference in cardiovascular risk between HOA and RA according to the Systematic Coronary Risk Evaluation 1 and SCORE2 models, except when the multiplication coefficient for RA was applied [45]. This suggests that the cardiovascular risk of HOA is similar to that of RA when RA is not considered as an independent cardiovascular risk factor. In our study, we found a similar risk of having a cardiometabolic disease between EHOA and RA, although RA was considered an independent cardiovascular risk factor. These results, showing non-different cardiovascular risks, do not reflect current practice where rheumatologists do not consider or underconsider the cardiovascular risk of HOA in contrast to RA patients. There are specific recommendations for managing cardiovascular risk in RA [44], while HOA is still considered by some rheumatologists to be a normal part of aging [18].

This study has several limitations. First, the age distributions differed between the RA and EHOA populations, which is inherent to the nature of the diseases. However, we stratified clinical outcomes by age group, and the findings in patients aged 51–70 years — the largest overlapping age group between the two populations — were consistent with those observed in the overall population. Secondly, the higher number of smokers in the RA group compared to the EHOA group can be attributed to the fact that smoking is a well-established risk factor for RA [46]. In contrast, alcohol consumption was less common in the RA group even though this variable was defined in the same way for both. Some evidence suggests that alcohol consumption may reduce the risk of RA [47], while data on its association with OA are contradictory. A meta-analysis found no association between alcohol consumption and OA risk after adjusting for confounding factors [48]. However, some studies suggest that alcohol consumption in patients with hip OA may increase the risk of needing a hip replacement [49]. Therefore, alcohol may be a factor involved in OA progression. Third, RA is a systemic disease affecting almost all joints, whereas HOA affects only the hand joints. However, it is rare for RA to be active without hand involvement, especially considering that pain assessment was global in these cohorts. Furthermore, the DIGICOD cohort includes patients with HOA, requiring at least two symptomatic joints for inclusion, which may overestimate the impact of HOA here. Nevertheless, the symptoms could be pain or nodal and to be included, patients did not have to meet a specific pain threshold, only to have experienced arthritis-related pain at some time point, thus excluding only radiographically asymptomatic OA. In fact, 13 ​% of patients had no painful joint on palpation at inclusion (and 12 ​% had only one) [12]. Conversely, the ESPOIR cohort has a very well structured follow-up which may not be generalizable to all RA patients. One of the limitations of our study is the loss to follow-up in the RA cohort at the 10-year visit. This may have led to an overestimation of disease severity in RA, as patients who remain in long-term follow-up tend to have more severe disease. However, this potential bias does not alter the overall conclusions of the study [50]. Our aim was not to compare two perfectly identical populations, but to compare the impact of two long-term and chronic diseases on the lives of patients, whose characteristics may differ.

Our study is the first to compare the burden of these two diseases, taking into account both the clinical impact of the disease on patients' lives and its associated comorbidities, with an adjustment for confounding factors. One of our strengths is the use of real-world data from two national prospective cohorts: ESPOIR and DIGICOD, which allowed the inclusion of nearly 500 patients. While scores in cohorts often are disease-specific scores, we were able to identify common outcomes enabling comparison between the two populations. In addition, we included patients with globally similar disease duration by selecting the 10-year visit for the ESPOIR cohort and the enrollment visit for DIGICOD, which is often differs in other studies. The median disease duration of included patients was 10.5 years, which allows to assess the impact of a chronic and long-standing disease.

In conclusion, our study shows that after more than 10 years of evolution, EHOA is associated with more pain and functional impairment than RA but with fewer comorbidities. This study highlights the significant unmet need for effective therapies for patients with EHOA.

## Author contributions

Conception and design: SB, ST, AR, BC, AS, LG, JS, AC.

Analysis and interpretation of the data: SB, ST, AR, NR, EM, BC, AS, BF, LG, FB, JS, AC.

Drafting of the article: SB, ST, AR, NR, EM, BC, AS, BF, LG, FB, JS, AC.

Critical revision of the article for important intellectual content: SB, ST, AR, NR, EM, BC, AS, BF, LG, FB, JS, AC.

Final approval of the article: SB, ST, AR, NR, EM, BC, AS, BF, LG, FB, JS, AC.

Provision of study materials or patients: NR, EM, BC, AS, BF, LG, FB, JS, AC.

Statistical expertise: ST, AR, NR, LG, AC.

Collection and assembly of data: SB, ST, AR, NR, LG, AC.

## Role of the funding source

None.

## Declaration of competing interest

Laure Gossec, disclosures outside the submitted work: research grants: AbbVie, Biogen, Lilly, Novartis, UCB; consulting fees: AbbVie, Amgen, BMS, Celltrion, Janssen, Lilly, MSD, Novartis, Pfizer, Stada, UCB; non-financial support: AbbVie, Amgen, Biogen, Janssen, MSD, Pfizer, UCB.
